# Spark Discharge Synthesis and Characterization of Ge/Sn Janus Nanoparticles

**DOI:** 10.3390/nano13101701

**Published:** 2023-05-22

**Authors:** Anna A. Lizunova, Vladislav I. Borisov, Dana Malo, Andrey G. Musaev, Ekaterina I. Kameneva, Alexey A. Efimov, Ivan A. Volkov, Arseny I. Buchnev, Ivan A. Shuklov, Victor V. Ivanov

**Affiliations:** 1 Moscow Institute of Physics and Technology, 141701 Dolgoprudny, Russia; 2 Biomedical Engineering Department, Faculty of Mechanical and Electrical Engineering, Damascus University, Damascus P.O. Box 30621, Syria

**Keywords:** germanium–tin nanoparticles, spark discharge synthesis, aerosol, thermal treatment, absorption, Raman spectroscopy, bicrystalline Janus nanoparticles

## Abstract

Germanium–tin nanoparticles are promising materials for near- and mid-infrared photonics thanks to their tunable optical properties and compatibility with silicon technology. This work proposes modifying the spark discharge method to produce Ge/Sn aerosol nanoparticles during the simultaneous erosion of germanium and tin electrodes. Since tin and germanium have a significant difference in the potential for electrical erosion, an electrical circuit damped for one period was developed to ensure the synthesis of Ge/Sn nanoparticles consisting of independent germanium and tin crystals of different sizes, with the ratio of the atomic fraction of tin to germanium varying from 0.08 ± 0.03 to 0.24 ± 0.07. We investigated the elemental and phase composition, size, morphology, and Raman and absorbance spectra of the nanoparticles synthesized under different inter-electrode gap voltages and the presence of additional thermal treatment directly in a gas flow at 750 °C. The research shows that the in-flow thermal treatment of aerosol-agglomerated nanoparticles produced special individual bicrystalline Janus Ge/Sn nanoparticles with an average size of 27 nm and a decreasing absorption function with a changing slope at 700 nm.

## 1. Introduction

Semiconductor materials compatible with silicon-based technology offer a challenge for materials science researchers to develop advanced devices and their applications. Nowadays, Ge/Sn is a promising and highly desirable material to extend the wavelength coverage well into the mid-infrared range in photonics, optoelectronics, and telecommunications [1,2], which can be used in lasers [3,4], photodetectors [5], solar cells [6], and sensors [7] operating at a wavelength higher than 1,1 µm at room temperature [1,2,3,4,5,6,7]. The growth of interest in creating Ge/Sn films is related to the elaboration of a direct-gap semiconductor with a tunable band gap in a broad range from near-hand zero to 0.67 eV [8,9,10]. Germanium is known to have an indirect energy band structure, which makes it complicated to acquire efficient light emission. Adding various contents of tin affects the energy band gap structure of the Ge_(1−x)_Sn_(x)_ semiconductors [10] and enables control of the lattice constant, band gap width, and subsequently, the optical properties of the material [11]. Since the edge of the conduction band for the direct (၃) conduction valleys decreases more rapidly than at the L-point (indirect valley), there is a certain critical value of tin concentration that yields the indirect-to-direct band-gap transition. Under different theoretical predictions, this transition occurs in a range of the Sn composition from 6 to 20% [8,10,11,12], which motivates researchers to achieve optimal conditions to engineer direct band-gap materials for functional devices.

Theoretical studies have demonstrated the potential emission in a range from 3 to 7 μm in strain-engineered self-assembled GeSn/GeSiSn quantum dots in the Ge matrix, and its suitableness for electrically pumped group IV lasers fully integrated into silicon [7]. Experimentally, Ge/Sn films and heterostructures have been produced with local concentrations of tin in a range from 2 to 28% [1,2,3,4,5,6,7,13,14]. Furthermore, it was shown that the optical absorption in films of the Ge_0.98_Sn_0.02_ alloy is ten times higher than that of pure germanium at a wavelength of 1550 nm and 20 times at a wavelength of 1620 nm [1]. 

Prototypes of lasers with electric and optical pumping functioning at temperatures of 90–270 K with emission peaks at 2.3–3.5 μm have been achieved on high-quality Ge/Sn films on germanium and silicon substrates by several groups [3,4,15,16,17]. The emission properties of Ge/Sn nanoparticles with segregated Sn nanoclusters on the surface of germanium nanoparticles [18], the usage of Ge/Sn layers for hybrid IR photodetectors [19,20,21] and adjustable optical modulators, functionalized from 1.8 to 5.0 μm [22,23], have also been demonstrated. 

The basic synthesis method of Ge/Sn films is known to be low-temperature or low-pressure molecular-beam epitaxy [1,2,3,4,5,6,7] and chemical vapor deposition from gaseous digermane (Ge_2_H_6_) and liquid tin tetrachloride [24,25]. Because of the low solid solubility of tin in germanium and the significant lattice mismatch between Sn and Ge, a number of requirements are imposed on these growth techniques for the creation of single crystalline Ge/Sn, specifically, a small temperature range for epitaxial growth, which can cause a breakdown [26], and the demand for a low temperature to provide Sn incorporation [1]. Moreover, Azrak E. et al. showed the production of core–shell fibers with 30% tin content [27] by low-temperature plasma-assisted growth. 

Ge/Sn nanoparticles were presented for use in Li-ion batteries, solar cells, and biosensors [28,29]. Moreover, the Ge_0.95_Sn_0.05_ nanocrystals of a 10 nm diameter exhibited a 25% higher capacity than Ge nanoparticles in anode materials [30]. A solution-based approach [31] proposes the synthesis of Ge/Sn alloy nanoparticles with a diameter of 3–25 nm and atomic tin content from 5 to 40% [32,33]. It was demonstrated that pulsed laser deposition and gas-phase laser photolysis allowed for producing Ge/Sn micro- and nanoparticles. In more detail, Ge_1−x_Sn_x_ nanocrystals produced by 1064 nm laser photolysis [30], with a 25% Sn content, were found to consist of different types of particles: biphasic Janus nanoparticles with β-Sn and cubic-phase Ge_0.75_Sn_0.25_ domains and separate β-Sn nanoparticles.

Promising applications of Janus nanoparticles have been suggested for biosensing, photocatalysis, drug carriers, and photonics [33,34,35] due to the combination of different physical and chemical properties in one nanoparticle. Several approaches have been engineered for the preparation of hard Janus biphasic nanoparticles composed of two different inorganic (hard) materials [36], including epitaxial deposition, a flame synthesis technique, phase separation techniques, and masking methods [37,38]. In recent years, nanostructured tin-based nanoparticles have been developed for different applications [39,40,41]. For instance, J. Li et al. [40] produced bimetallic colloid NiSn nanocrystals for electrocatalysis by the co-reduction of two metals. In addition, CoSn/Co_3_Sn_2_@C materials with a nanocage structure were fabricated as anodes for lithium-ion batteries [41].

An alternative technology for alloy synthesis from immiscible materials, proposed in this work, is the spark discharge synthesis of aerosol nanoparticles [42,43]. Using this method, nanoparticles of the metal alloys of Ag-Cu, Au-Pt, Cu-W [44], and InSn [45] have been successfully produced. The theoretical models and supporting experimental studies presented in [46,47] have described the possibility of controlling the elemental composition of nanoparticles Au/Pt and Au/Ag by varying the parameters of the current waveform and their polarity, while Au, Ag, and Pt were reported as materials with a closed coefficient of erosion under an electrical current. Furthermore, complex multiple-element nanoparticle alloys containing up to six materials were prepared by spark discharge [48] in 2020. In addition, the studies dedicated to the synthesis of nanoparticles of pure semiconductor nanoparticles by spark discharge succeeded in obtaining crystals of germanium and silicon in an amorphous matrix [49,50]. Core–shell nanoparticles with a tin core and complex Ge/Sn oxide shell [51] and a Cu core and Ag shell [52] were also produced by the co-sparking method. The first attempts at generating nanoparticles with a Janus morphology and biphasic/bicrystalline type by spark discharge were presented at the beginning of 2023 by the Mo_3_Ni_2_N case [53]. The additional system of the in-flow sintering of agglomerates formed in the spark discharge was used to reshape agglomerates into separated large nanoparticles and can lead to the segregation of some phases [52]. Relating to Ge/Sn, an increase in the synthesis temperature and additional annealing of Ge/Sn films above the critical temperature cause the segregation of the tin into separate Sn-rich islands. Tin segregation occurs at different temperatures varying from 275 to 750 °C, depending on the tin content and the method of production of Ge/Sn films [54,55,56]. The segregation process improved the crystalline quality of Ge/Sn films and increased the responsibility of p-i-n photodetectors based on annealed Ge_0.90_Sn_0.10_ films at 1550 nm by 33% in comparison to as-grown ones [55]. 

Notably, an alloy is an extensive term defining a material containing two or more elements existing together in a different manner. In this article, we prefer to use the term alloy for a homogeneous mixture (solid-state solution) of two materials, where one chemical component is substituted for another in a crystallographic-ordered periodic lattice [57]. We use the definition of heterogenous bicrystalline material for a material in which two different crystal phases exist together and are completely insoluble. 

Inspired by the latest developments in spark discharge techniques and the promising outlook of Ge/Sn nanomaterials, this research aims to extend the limits of spark discharge to create mixed nanomaterials with extremely huge differences in their electrical erosion rates, such as tin and germanium. The second aim is to investigate the types of germanium–tin nanoparticles one can produce by spark discharge and establish the dimensional, structural, elemental, and optical properties of the obtained nanoparticles, depending on the parameters of the spark discharge synthesis.

## 2. Materials and Methods

**Figure 1:**
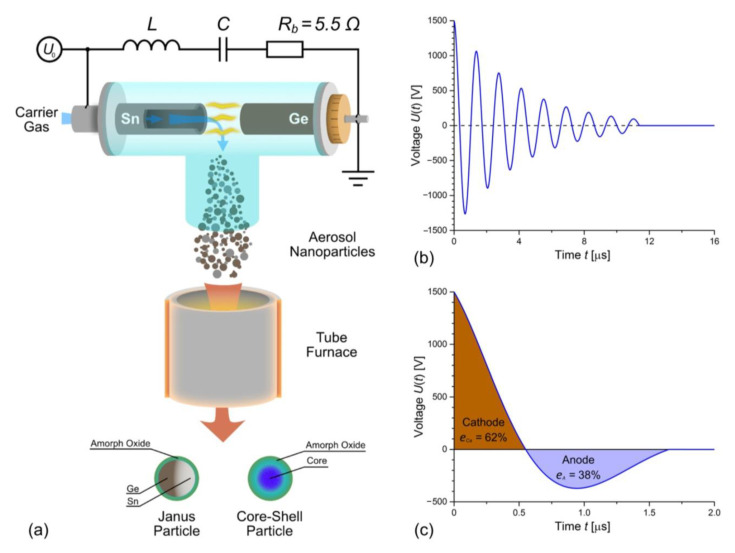
(**a**) Scheme of the gas-phase synthesis of Ge/Sn nanoparticles in SDG, oscillograms of spark discharge voltage U(t) measured between Sn and Ge electrodes during a spark discharge (**b**) without the ballast resistor and (**c**) with the ballast resistor R_b_ = 5.5 Ω.

A schematic representation of a spark discharge generator for the synthesis of aerosol Ge/Sn agglomerates and a custom base tube furnace for the subsequent in-flow agglomerate sintering are shown in Figure 1. To produce a general mixture of Ge/Sn primary nanoparticles by co-sparking, an ignition between Sn and Ge electrodes was created. As a result of the discharge between the Sn and Ge electrodes, their simultaneous erosion occurred. As both materials evaporated, a mixed vapor was formed in the interelectrode gap. Then, the vapor cooled down and condensed into primary nanoparticles with a mixed composition. The nanoparticles, captured by the carrier gas flow, formed aggregates and agglomerates of different sizes until they finally cooled down. For the in-flow conversion of the agglomerates into nanoparticles of different morphologies and sizes, a tube furnace with a temperature that can be adjusted from 25 to 1000 °C was used. General agglomerated nanoparticles passed through the furnace heated to a dedicated temperature, then melted and formed larger particles with a spherical shape under emerging surface tension forces.

To prevent oxygen and water diffusion through the walls and connections, the gas path of the SDG was constructed from stainless steel and glass using the standard KF vacuum fittings. The discharge chamber was a DN-50-sized T-shaped Millan Duran glass, into which two electrode holders were mounted; see Figure 1a. The cathode holder was equipped with a micrometer screw with a linear step of 50 μm to control the distance of the discharge gap. The anode holder had a coaxial channel to supply the carrier gas in the discharge gap. This supply configuration of the carrier gas ensured the efficient removal of nanoparticles from the discharge gap. Tin and germanium rods of 8 mm in diameter and 30 mm in length were used as an anode and a cathode, correspondingly. The Sn anode had a coaxial hole with a diameter of 3 mm for gas passage. Prior to any experiments, a medium vacuum of about 0.4 Torr was maintained by a foreline pump in the gas path for 30 min. An argon carrier gas with a purity of 99.9999% (6.0) was supplied to the discharge gap at a flow rate of 1 L/min, controlled by an SMC PFM2-SP flow meter. The gas pressure in the discharge chamber was maintained at approximately 1.3 bar.

The as-generated Ge/Sn heterogeneous agglomerates were subsequently moved to thermal treatment in the tube furnace heated to 325 and 750 °C. The generated nanoparticles underwent sintering for 18 s. This time was estimated by relying on the gas flow rate, the gas path cross-sectional area, and the length of the furnace, which were 1 L/min, 4.9 cm^2^, and 60 cm, respectively. In our experiments, the Ge/Sn mixed nanoparticles produced without using the tube furnace (initial as-generated agglomerates) and obtained after sintering at 325 and 750 °C were named samples 25, 325, and 750 °C, correspondingly. The temperature of 325 °C was chosen for the experiments based on the literature as the condition that can provide the production of a solid-state solution of Ge/Sn alloy [24,25,28]. According to Li et al. [58], Ge/Sn alloy sintering below 400 °C remains a solid-solution alloy, whereas decreasing the temperature results in the formation of Sn-rich clusters and layers. The temperature of the Sn surface segregation depends on the thickness of the Ge/Sn layer and decreases with increasing Sn content, so the segregation process can partly start at temperatures as low as 275 °C [56]. At 750 °C, the morphology of Ge/Sn nanoparticles is suggested, converting from agglomerates into individual nanoparticles as the bare germanium agglomerates synthesized in the spark discharge are reshaped into individual nanoparticles at close values of temperature [59].

A spark discharge was initiated by applying a breakdown voltage *U*_0_ to the electrodes through a capacitor *C*, charged from a high-voltage source; see Figure 1a. The capacitor was assembled from ZVEK PROGRESS KVI-3 4700 pF low-inductance high-voltage pulse capacitors connected in parallel. The total capacitance of the capacitor was 38 nF. The electrical circuit had parasitic inductance *L* and resistance, which were 1.27 μH and 0.6 Ω, respectively. The spark gap distances, discharge voltages, and discharge repetition rates were 0.8 mm and 3.3 mm, 1.5 kV and 3.0 kV, and 550 Hz, respectively. The spark discharge voltage *U(t)* was measured with a custom capacitive-resistive voltage divider connected in parallel with the capacitor. The signal from the voltage divider was given to a Tektronix DPO 4102B-L oscilloscope.

In common SDG configuration, the oscillations had a low damping (Figure 1b), and the anode Sn and cathode Ge dissipated energy rates were almost equal. The fraction of the discharge energy released at the cathode and anode can be calculated from the current (voltage) oscillograms [47]. In the case of using a low-damped electric circuit in our experiments, the share of energy falling on the cathode was 55%, and on the anode, it was 45%. Under these conditions, approximately 95% of the obtained crystals in the produced nanoparticles were *β*-tin [51]. 

It is known that the property of a material to ablate under the electric current propagation can be described by L.S. Palatnik’s criterion [60], who proposed to estimate the kinetics of the mass transfer of electrode materials, considering their density ρ, heat capacity C_m_, coefficient of thermal conductivity λ and melting point T_m_, by using the following formula:

(1) $$K=C_{m}{\rho \lambda T}_{m}^{2},$$

where K is the erosion resistance of the material (proportional to the time of melting of a certain volume of the material).

The Palatnik criterion is also called a physical coefficient of resistance to erosion and is proportional to time, so it indicates which of the materials sooner or later heats up to the melting point. According to the thermal theory of erosion of the electrode material, the higher K, the more energy must be spent on erosion per unit mass of the material, i.e., the worse (slower) the erosion process occurs. The erosion resistance (K) of germanium is 5.5 times higher than that of tin, which means that the thermal erosion of germanium occurs 5.5 times slower than that of tin.

Another way to estimate the ability of an electrode to erode was suggested by Feng in 2018 [47], who described the criterion to ablate with the use of the enthalpies of the melting H_m_ and vaporization H_e_ (J kg^−1^) points, the heat capacity, and the boiling point of the material:

(2) $$C_{m}=\left(C_{\mathrm{ps}}\left(T_{b}-T_{c}\right)+H_{m}+H_{e}\right),$$

where C_ps_ (JK^−1^ kg^−1^) is the heat capacity of the solid material, T_c_ and T_b_ are the temperatures of the carrier gas and the boiling point of the electrode materials, correspondingly, whereas H_m_ and H_e_ are their enthalpies of melting and vaporization.

The ratio of the constants C_m_ calculated by Feng’s formula for germanium and tin is 5.3; this gives identical results to the Palatnik criterion and indicates a high difference in the ability to erode under the influence of an electric current for electrodes made from tin and germanium.

Thus, if we use a low-damped electrical circuit with the simultaneous erosion of germanium and tin electrodes, in which 55% spark energy is released on the cathode, we can only obtain an alloy with a mass fraction of tin approximately five times higher than the germanium. We demonstrated this effect in our previous work [51], when we produced core–shell nanoparticles with a *β*-Sn core and complex germanium–tin oxide shell with an average mass content of tin of more than 70%. This means that the creation of an alloy semiconductor nanomaterial with a tin content of less than 30% is necessary to alter the electric circuit of the spark discharge generator in such a way that 20% of the energy will dissipate on the tin anode.

The anode Sn production rate can be decreased by increasing the active resistance of the circuit. Assuming that the SDG electrical scheme is an RLC circuit with time-invariant RLC parameters, the critical circuit resistance *Rc* = 11.6 Ω was calculated from the equality condition of the damping coefficient *δ* and the natural frequency ω_0_:

(3) $$R_{c}=2\sqrt{\frac{L}{C}},$$

where *L* is the inductivity, and *C* is the capacitance.

If the circuit resistance *R* = *R_c_*, the anode Sn dissipated energy was too small, and the synthesized Ge/Sn nanoparticles were predominantly germanium. The ballast resistor Rb = 5.5 Ω was experimentally chosen to obtain Ge/Sn nanoparticles in almost equal proportions of Sn and Ge. In this case, the energy percentages e of the anode Sn and cathode Ge were 19.7 and 80.3%, respectively; see Figure 1c.

The particle size distribution, morphology, crystal structure, and element composition of the nanoparticles were investigated using the JEOL JEM-2100 transmission electron microscope (TEM) with the energy dispersive X-ray (EDX) spectrometer from X-MAXN OXFORD Instruments. For the microscopic studies, a TEM grid with carbon film was mounted on a HEPA filter to collect nanoparticles in the gas path. The average values of the particle sizes, interplane distances, and atomic compositions are presented with an absolute standard deviation, which was calculated as the square root of the variance of the corresponding dataset. The real-time measurements of the particle size distribution in the aerosol flow were determined by the TSI SMPS 3936 aerosol spectrometer. The mass production rate of the synthesis of the nanoparticles was estimated by the gravimetric method using the Sartorius Secura 225D-1ORU analytical balance.

The UV-vis-NIR extinction spectra of the nanoparticle dispersions in a quartz cuvette were measured using the JASCO V–770 spectrophotometer. To study the obtained nanoparticles using the spectrophotometer, the nanoparticles were precipitated from the HEPA filter and diluted with isopropyl alcohol (LiChrosolv, Merck, Darmstadt, Germany), and then sonicated for three minutes. Crystalline phase analysis of the bicrystalline nanoparticles collected on the filter was carried out by X-Ray diffraction analysis (XRD) using the Thermo Scientific ARL X’TRA X-Ray diffractometer equipped with a parabolic mirror (AXO Dresden, Dresden, Germany) and a pinhole collimator. Fourier transform infrared (FTIR) spectra in an attenuated total reflection mode were recorded on a Nicolet™ iS50 FTIR Spectrometer (Thermo Scientific, Waltham, MA, USA) directly from the nanoparticles on the cellulose filter. Raman spectroscopy studies were performed using the DXR Raman Microscope (Thermo Scientific, Waltham, MA, USA) with a 532 nm laser (0.1 mW).

## 3. Results and Discussion

### 3.1. TEM Results

**Figure 2:**
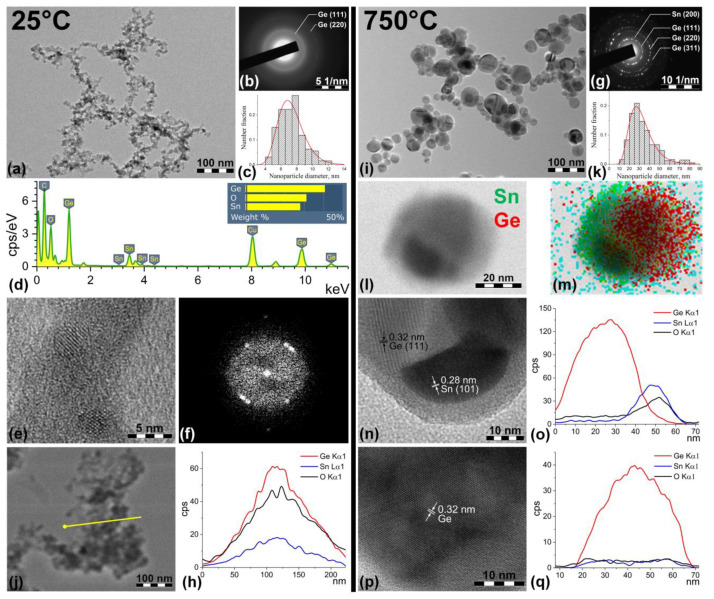
TEM analysis of the obtained Ge/Sn nanoparticles by spark discharge without thermal treatment (25 °C, 1.5 kV, left column) and with additional in-flow heating in the furnace at 750 °C, 3 kV (right column). Typical TEM images (**a**,**i**) and the corresponding diffraction patterns (**b**,**g**) of the Ge/Sn agglomerates, synthesized at 25 °C and 750 °C; (**c**,**k**) primary particle size distribution, approximated by a lognormal function, (**d**) EDX spectrum of the agglomerate, (**e**) high-resolution TEM image, and (**f**) corresponding FFT pattern of germanium crystallites in an amorphous matrix; (**j**) scanning TEM image and the line along which the EDX profile was scanned and (**h**) EDX element profile of the agglomerate; (**l**,**m**) elemental map of Janus Ge/Sn nanoparticle, where red color represents the position of Ge, and green color for Sn; (**n**,**o**) HRTEM image, and EDX elemental profile of the Janus bicrystalline core-shell nanoparticle synthesized at 750 °C; (**p**,**q**) HRTEM image and EDX elemental profile of the pure core germanium nanoparticle with an oxide germanium–tin shell.

TEM images of the Ge/Sn nanoparticles produced by spark discharge without using a furnace in the gas path and with additional thermal sintering at 750 °C are presented in Figure 2, and they show the different morphologies of the particle ensembles. According to the TEM images, the primary particles produced in the spark discharge formed fractal-like agglomerates, which consisted of small spherical crystals in an amorphous matrix (Figure 2e). The number-average size of primary particles prepared at different breakdown voltages and a furnace temperature of 25 °C is basically identical and equal to 7.4 ± 1.6 and 7.5 ± 1.8 nm for the breakdown voltages of 1.5 and 3.0 kV, respectively. We analyzed 149 and 290 primary nanoparticles for the 1.5 and 3.0 kV samples, respectively, to construct the particle size distribution and calculate the average diameters and their standard deviations. The particle size distribution is well described by a lognormal function and shown in Figure 2c. The lognormal distribution is typical of nanoparticles produced by physical synthesis methods as a gas discharge [61,62]. The selected electron diffraction (SAED) patterns contain two concentric circle reflexes, which are a superposition of faded point diffuse reflections from small-disoriented crystallites, with the interplanar distance close to that of cubic germanium. The indicated SAED circle reflexes (Figure 2b) belong to the interplanar distances of 0.320 ± 0.004 nm and 0.197 ± 0.004 nm, which correspond to the (111) and (220) planes of pure Ge (Fd3m), respectively. The high-resolution TEM images confirm the SAED data and explain the morphology of the agglomerates as crystals of germanium with sizes of 3–6 nm in diameter in an amorphous matrix. 

The overall weight composition of Sn and Ge was investigated using EDX analysis. The average ratio of the atomic fraction of tin to the atomic fraction of germanium and the corresponding standard deviations are represented in Table 1. The as-generated nanoparticles contained germanium, tin, and oxygen; the atomic fraction of oxygen was about 66–68% regardless of the gap breakdown voltage (Figure 2d). It was found that the composition of tin and germanium was inhomogeneous and varied among the agglomerates. We analyzed approximately 15 agglomerates for each sample. The nanoparticles prepared at a voltage of 1.5 kV without additional thermal treatment were characterized by the following average compound: tin—3.8 At. %, germanium—27.9 At. %, and 68.3 At. % of oxygen. With respect to the atomic composition, the average ratio of the atomic fraction of tin to the atomic fraction of germanium was calculated as 0.14 ± 0.03. A twofold increase in the voltage from 1.5 to 3.0 kV led to a notable growth of the tin content in the nanomaterial, and the average ratio of the atomic fractions of tin to germanium was observed to be 0.24 ± 0.07 for the nanoparticles synthesized at 3.0 kV against 0.14 ± 0.03 for 1.5 kV.

**Figure 3:**
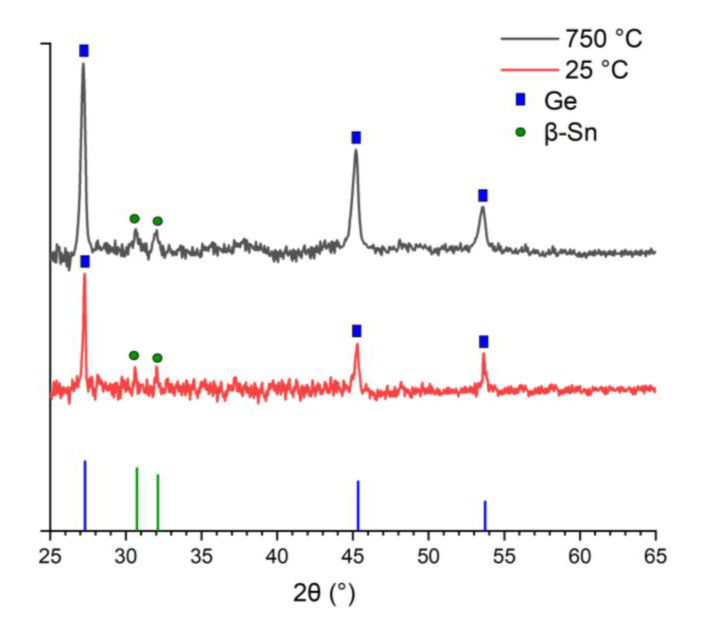
XRD patterns of the Ge/Sn nanoparticles prepared as generated by spark discharge (red line) and with additional heat treatment in a tube furnace at 750 °C (black line).

According to the FTIR data, the intensive absorption bands at ~542, ~809, and ~1636 and a broad peak from 3000 to 3600 cm^−1^ with a maximum at 3225 cm^−1^ were detected for the as-generated agglomerates and nanoparticles after thermal sintering at 325 °C. Only diffuse traces of these peaks were detected for the nanoparticles prepared after high-temperature (750 °C) sintering. Notably, the Ge-O bands were always located at 566 and 820 cm^−1^ [63], and the peaks at positions 608–675 were attributed to the Sn-O-Sn band [64,65]. The positions of the obtained FTIR bands for the heterogeneous agglomerates Ge/Sn were close to the Ge-O and Sn-O bands but non-identical. A strong O-H stretching peak at ~3225 cm^−1^ was assigned to the overlapping O-H stretching mode of water (~3100 cm^−1^) and the surface Ge-OH, Sn-OH, or Ge/Sn-OH bands. A much weaker band around 1636 cm^−1^ was also attributed to H-O-H bending. The XRD patterns represented in Figure 3 for the as-generated Ge/Sn agglomerates show intensive reflexes of the cubic germanium and weak reflexes of the β-tin. Thus, relying on the FTIR, EDX, XRD, and TEM data, we produced heterogeneous Ge/Sn agglomerates containing separate predominantly germanium crystals and few tin crystals in a complex germanium–tin oxide amorphous matrix for the 25 and 325 °C samples. 

The morphology of generated agglomerates with subsequent sintering at 325 °C was similar to the initial agglomerates (see Supplementary Figure S1), but the average size of the primary nanoparticles increased to 11.3 ± 2.7 nm, which was connected to the partial melting of tiny nanoparticles in the furnace and the beginning of the morphology change process [59,60]. 

The as-generated aerosol agglomerates of Ge/Sn obtained from the spark discharge were moved to a tube furnace heated to 750 °C in 18 s by the gas flow. The size of the aerosol nanoparticles, measured by aerosol spectroscopy, decreased from 202 to 95 nm after moving through the furnace heated to 750 °C. This phenomenon occurred due to the melting of primary particles in the agglomerates. They were reshaped in liquid form into a well-rounded drop and then further recrystallized into compact spherical crystal nanoparticles. The temperature of the complete reshaping of the agglomerates into spherical particles is a function of the primary particle diameter in the agglomerates and the thermal properties of the material of electrodes, and it is lower than the melting point of the appropriate bulk material, which has been demonstrated earlier in experiments with silver and germanium nanoparticles [59,66]. The TEM images represented in Figure 2 show a morphology change from fractal-like agglomerates to large individual particles with a wide range of diameters varying from 9 to 89 nm for a gap voltage of 1.5 kV and from 12 to 82 nm for 3 kV. The average sizes of the individual particles did not depend on the voltage and were equal to 29.7 ± 12.1 and 33.8 ± 13.7 nm for breakdown intergap voltages of 1.5 and 3.0 kV, correspondingly. We processed images of 324 and 550 individual nanoparticles for the 1.5 and 3.0 kV samples, respectively, to construct the particle size distributions.

The results of the EDX studies reveal a decrease in the relative composition of Sn when the temperature of the sintering increased. This fact can be explained by the partial evaporation of tin from the surface of the heterogeneous agglomerated nanoparticles. A similar effect was discussed in the study of spark discharge synthesis of mixed AuGe aerosol agglomerates [67]. As the melting point temperature of tin is significantly lower (232 °C) than for germanium (938 °C), the loss of mass of tin from the surface of the agglomerates by evaporation was observed in the 325 and 750 °C samples in contrast to the nonevaporated germanium. So the relative atomic composition (Ge/Sn) of the as-generated agglomerates (25 °C sample) prepared at 1.5 kV was approximately 75% higher than for the 750 °C sample that underwent the in-flow thermal treatment.

The electron diffraction patterns for the nanoparticles prepared with additional heat treatment contained bright point reflexes related to the crystalline phase of the cubic germanium and β-tin. The only non-overlapping SAED reflexes for these two phases were rings attributed to the *d*-spacings of 0.321 ± 0.004 nm (Ge, plane (111)) and 0.275 ± 0.006 nm (β-Sn, plane (101)). The obtained values of the interplanar distances are fully confirmed by the X-ray diffractometry analysis presented in Figure 3 for the 25 and 750 °C samples, showing the presence of only the Ge and β-Sn peaks. The XRD pattern for 325 °C had similar signals and is demonstrated in Supplementary Figure S2. It is known that Sn incorporation into a Ge lattice results in a 0.05–1 % shift in the XRD peaks of Ge to low angles and the appearance of an extra peak in the position of 2θ ≈ 65°, corresponding to longer Ge/Sn bonds [67,68]. In our experiments, neither the shift nor the 65° peak occurred, which indicates the absence of a solid solution of Ge/Sn or its insignificant concentration due to the limitation of the X-ray diffractometric analysis, in which the sensitivity to the concentration of crystal impurities was 2–5 mass%. No solid solution of Ge/Sn was observed, i.e., neither for the nanoparticles undergoing thermal treatment nor without it. 

According to the high-resolution TEM images, different crystal types of nanoparticles were observed after the thermal treatment of Ge/Sn agglomerates in the tube furnace at 750 °C: firstly, the core–shell bicrystalline Janus nanoparticles; secondly, core–shell nanoparticles with a pure cubic germanium core. 

The first type of nanoparticles represents the heterogeneous core, consisting of two crystals: a massive cubic germanium nanocrystal and a small crystallite of β-tin (Figure 2l,n), which was confirmed by the EDX elemental profiles and maps within one particle. The thickness of the amorphous shell, whose element composition was suggested to be germanium–tin oxide, varied from particle to particle, with a range from 2 to 7 nm. Secondly, few particles with a pure germanium core and a complex oxide shell were found (Figure 2p,q). That is, during the heat treatment, redundant oxygen was burning out, the tin segregated into a separate crystal, and the introduction of tin atoms into the atomic lattice of germanium did not occur. The average elemental composition of individual Ge/Sn nanoparticles was determined by energy dispersive analysis; as a result, the average atomic ratio of tin to germanium was 0.08 ± 0.03 for 1.5 kV and 0.19 ± 0.07 for 3.0 kV. The atomic fraction of oxygen in the particles was less than 37 At. % (that is 10 Wt. %) and was probably located in the thin amorphous shell on the surface of the particles (Figure 2n). In addition, as in the case without the furnace, the atomic fraction of tin increased with increasing voltage across the gap. We attribute this effect to the low melting point of tin, which led to rapid heating and melting of the tin surface with the increase in the voltage, and therefore, more material evaporated from its surface into the interelectrode gap, in contrast to the germanium. Thus, using additional sintering at 750 °C, one can produce heterogeneous bicrystalline Ge/Sn with separate germanium and β-tin crystals with an interface region. 

### 3.2. Raman Spectroscopy

It is known that the Raman frequency shift in alloy nanoparticles is influenced by crystal defects and associated with the particle size, crystal phase, and local element composition [67,68,69,70,71]. Studies published in 2017–2018 showed the relationship between the local environment of Ge atoms in Ge/Sn alloys and the characteristics of the bond strain and lattice deformation [69,70]. The results obtained made it possible to evaluate the composition of the obtained Ge/Sn alloys from the shift of the Raman peaks of the Ge/Sn samples. In this paper, we assumed that the vibrations in the crystal lattice of bicrystalline particles and particles in the matrix can be described by a model of a solid-state alloy in thin films. The atoms of tin and germanium located in the phase interface of Janus Ge/Sn NPs were close to each other, as in the alloy films. On the other hand, surface atoms also contribute to the oscillations of the optical phonons due to the small size of Janus nanoparticles. So, based on the general physical principles of Raman spectroscopy, we decided to calculate the nanoparticles’ composition using the thin-film alloy theory.

According to the equation derived in [71], the Raman shift for the Ge-Ge bond in the strained solid solution containing more than 0.1% tin can be estimated by the following linear function:

ω_Ge-Ge_= ω_0_ ± 95·x [cm^−1^] (4)

where ω_Ge-Ge_ is the peak position of the Ge-Ge phonon band in the experimental spectrum, ω_0_ is the Raman peak for the unstrained Ge crystal at a temperature of 300 K located at 300.9 cm^−1^, and x is the tin range content.

It was found that the frequency of optical phonons localized in the nanocrystals increased with the growth of the crystal’s dimensions [72,73] up to a certain limit when achieving the value of bulk material. In the work by [74], a phonon confinement model to analyze the Raman spectra of germanium nanocrystals was developed and experimentally confirmed. The research showed a decreasing effect of the Raman shift to lower wavenumbers of 15 cm^−1^ with a decrease in the size of the germanium crystal from 10 nm to 1 nm. For crystal sizes, an optical phonon frequency of more than 10 nm is identical to the bulk material. As we detected by HRTEM, the size of the obtained germanium crystals was approximately 4 nm, with the Raman peak according to the model by [74] being in the position of 296.5 cm^−1^. 

**Figure 4:**
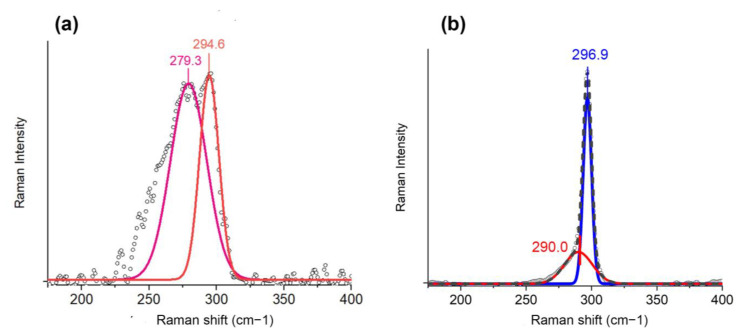
Measured (dots) and approximated (lines) Raman shifts for the Ge/Sn nanoparticles: (**a**) as-generated agglomerates prepared without heat treatment, (**b**) Janus bicrystalline nanoparticles formed after in-flow heat treatment at 750 °C.

Typical Raman spectra of the heterogeneous bicrystalline Ge/Sn agglomerates prepared as generated by spark discharge are shown in Figure 4a. The Raman shifts were detected in the positions of 280.0 and 279.3 cm^−1^ for the samples, synthesized at gap voltages of 1.5 and 3.0 kV, respectively. The calculation of the tin content using Equation (4) and ω_0_ = 296.5 cm^−1^ for the obtained Ge/Sn nanoparticle agglomerates prepared at 25 °C with the morphology of the agglomerates gave estimations of the ratios of the atomic fractions of tin to germanium Sn/Ge = 0.20 (1.5 kV) and 0.22 (3 kV), which are comparable with the EDX data. The implicit Raman peak in the position of 296.4 cm^−1^ in Figure 4a is attributed to the main Ge-Ge bond mode for the nanosized bare germanium crystals.

For the Janus nanoparticles produced by spark discharge with additional in-flow sintering at 750 °C, the Raman peak (Figure 4b) was approximated by the sum of two Gaussian curves with positions at 290.0 and 290.8 cm^−1^, which characterizes the Ge-Ge_DA_ bond mode activated by structural heterogeneity, and at 296.9 and 297.2 cm^−1^, which characterizes the main Ge-Ge bond mode for the samples synthesized at 3.0 and 1.5 kV, respectively. Comparing the measurement results with the given ones in Equation (4), the estimated tin contents in the samples obtained at 750 °C were 0.039 and 0.042 for 1.5 and 3.0 kV, respectively. A subtle, hardly visible Ge/Sn (~265 cm^−1^ [75]) mode was observed for the Janus bicrystalline nanoparticles, which was likely related to the interface between the germanium and tin crystals in one nanoparticle. So, essentially, the correlation of the XRD data with the electron diffraction patterns proves that solid-solution Ge/Sn alloy nanoparticles were not formed by spark discharge. 

### 3.3. Optical Properties

**Figure 5:**
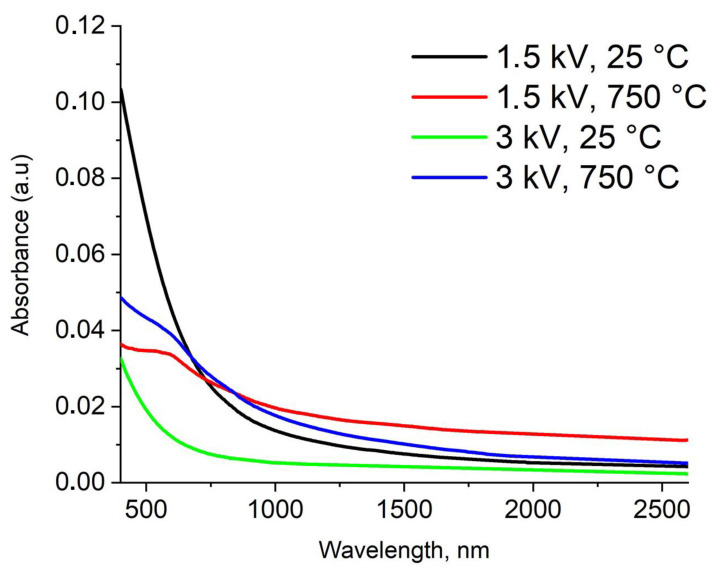
Absorbance spectra of Ge/Sn NPs.

The absorption spectra of the obtained heterogeneous Ge/Sn nanoparticles are presented in Figure 5 and show an optical density increase with a wavelength decrease. The absorbance was a uniformly decreasing function of wavelength for the agglomerates and a descending function with a changing slope for the Janus particles, for which the absorbance reflection observed was approximately at 700 nm. Almost uniformly, the UV-vis-NIR spectra obtained in our experiments for the mixed agglomerates and the Janus NPs are close to the measured ones for the Ge/Sn alloy nanoparticles produced by colloidal green synthesis and laser ablation with modification in previous research [76,77].

Notably, a complex function of the physical parameters, such as size, composition, shell thickness, and morphology, determines the optical properties of nanoparticles. It is almost impossible to separate the influence of one property from another. It is known that increases in the particle size, shell thickness, and nanoparticle agglomeration lead to a red shift in the particles’ absorption [78,79,80,81]. On the other hand, the enlargement of the particle size distribution of metal and semiconductor nanoparticles can result in the appearance of additional peaks in the longer wavelength region and the broadening of available ones [51,81]. 

## 4. Conclusions

The developed modification of a spark discharge generator damped for one period allowed for the creation of novel Janus nanoparticles from heat-resisting and low-melting metal materials with unique morphological and optical properties, which can be used to form infrared devices and plasmonic structures.

As-generated agglomerates of predominantly germanium nanocrystals in a complex amorphous matrix and individual bicrystalline Janus Ge/Sn nanoparticles with average sizes from 7 to 30 nm were produced. The obtained Janus nanoparticles consisted of germanium and tin crystals with an interface region, and the ratio of the atomic fraction of tin to germanium varied from 0.08 ± 0.03 to 0.19 ± 0.07. The ratio of the atomic fraction for the as-generated Ge/Sn agglomerates ranged from 0.14 ± 0.03 to 0.24 ± 0.07. The optical properties of the obtained nanoparticles depended on the morphology, composition, and size of the nanocrystals. It is estimated that the absorption spectra of the individual Janus nanoparticles are a descending function with a changing slope at 700 nm.

The obtained nanoparticles can be used as a source of aerosol nanoparticles to fabricate films and microstructures using dry aerosol printing technology [82], and for preparing conductive nano-inks [83] for printed electronics to create chemical and biological sensors, infrared photodetectors, and diodes. 

## Figures and Tables

**Table 1:** Influence of sintering temperature on the elemental composition of the Ge/Sn nanoparticles. Averaged ratios of atomic fraction of tin to germanium and the corresponding absolute standard deviations for the obtained samples are represented. The numbers of analyzed agglomerates are denoted in the brackets.

Sample	Atomic Composition Ge/Sn, 1.5 kV	Atomic Composition Ge/Sn, 3 kV	Presence of Oxygen, O, At. % (3 kV)
25 °C	0.14 ± 0.03 (11)	0.24 ± 0.07 (13)	66
325 °C	-	0.21 ± 0.06 (12)	70
750 °C	0.08 ± 0.03 (19)	0.19 ± 0.07 (17)	37

## Data Availability

All data generated or analyzed during this study are included in this published article (and its Supplementary Materials file). The datasets of the XRD patterns analyzed during the current study are the mix of known crystallographic structures of bulk Ge and white tin, which are available, for example, at the American Mineralogist Crystal Structure Database (http://rruff.geo.arizona.edu/AMS/amcsd.php, accessed on 4 June 2019).

## Supplementary Materials

The following supporting information can be downloaded at: https://www.mdpi.com/article/10.3390/nano13101701/s1, Figure S1: (a) TEM image of the Ge/Sn agglomerates prepared by spark discharge with additional thermal treatment at 325 °C, and (b) the corresponding primary particle size distribution, approximated by a lognormal function; Figure S2: The XRD pattern of the Ge/Sn agglomerated nanoparticles prepared by spark discharge with additional heat treatment in a tube furnace at 325 °C; Figure S3: TEM images of the individual Ge/Sn nanoparticles with crystal core covered by amorphous shell, prepared by spark discharge with additional heat treatment in a tube furnace at 750 °C.
