# Supplementary material for: Spark Discharge Synthesis and Characterization of Ge/Sn Janus Nanoparticles
Source: Nanomaterials (Basel). 2023 May 22;13(10):1701. doi: 10.3390/nano13101701 (PMC10223582; doi:10.3390/nano13101701)
Supplement: Supplementary file 1 [file nanomaterials-13-01701-s001.zip › nanomaterials-2367770-supplementary.pdf]

Supplementary Information

Spark Discharge Synthesis and Characterization of Ge/Sn Janus Nanoparticles

Anna A. Lizunova [1,*], Vladislav I. Borisov [1], Dana Malo [1,2], Andrey G. Musaev [1], Ekaterina I. Kameneva [1], Alexey A. Efimov [1], Ivan A. Volkov [1], Arseny I. Buchnev [1], Ivan A. Shuklov [1] and Victor V. Ivanov [1]

[1] Moscow Institute of Physics and Technology, 141701 Dolgoprudny, Russia
[2] Damascus University, Biomedical Engineering Department, Faculty of Mechanical and Electrical Engineering, P.O.Box 30621, Damascus, Syria
* Correspondence: lizunova.aa@mipt.ru

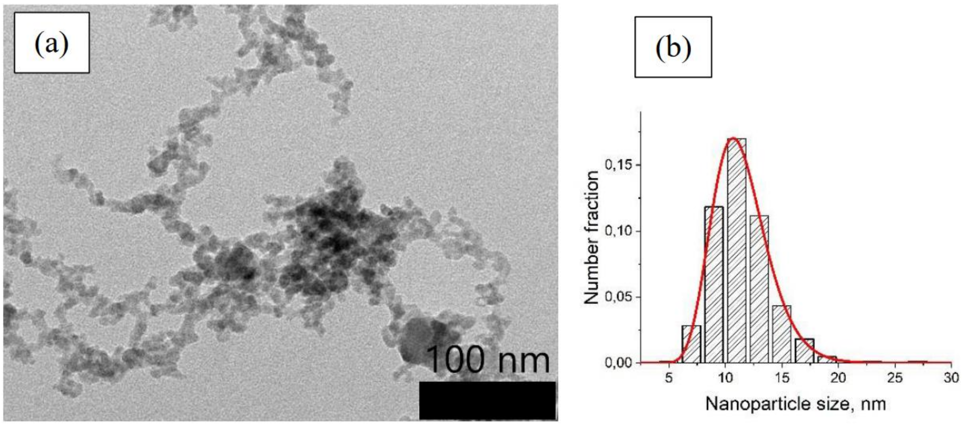

Figure S1. (**a**) TEM image of the Ge/Sn agglomerates prepared in spark discharge with additionalthermal treatment at 325 °C and (**b**) the corresponding primary particle size distribution, approximated by a lognormal function.

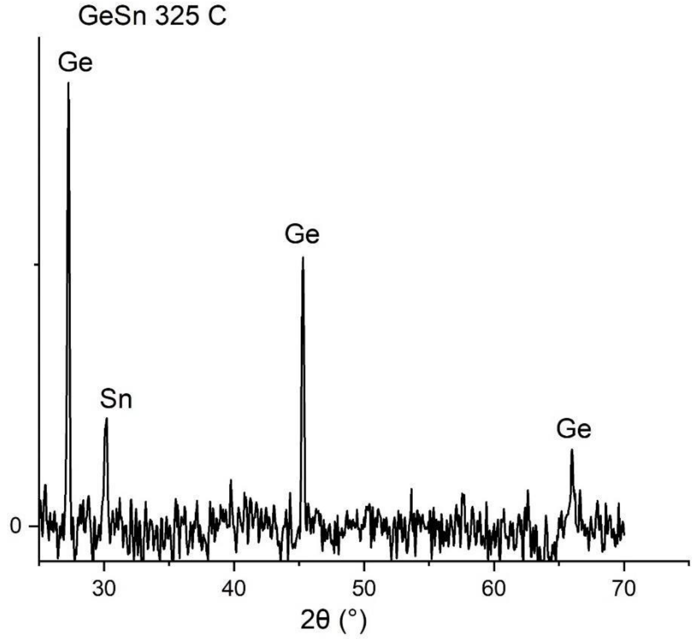

Figure S2. The XRD pattern of the Ge/Sn agglomerated nanoparticles, prepared in sparkdischarge with additional heat treatment in a tube furnace at 325 °C.

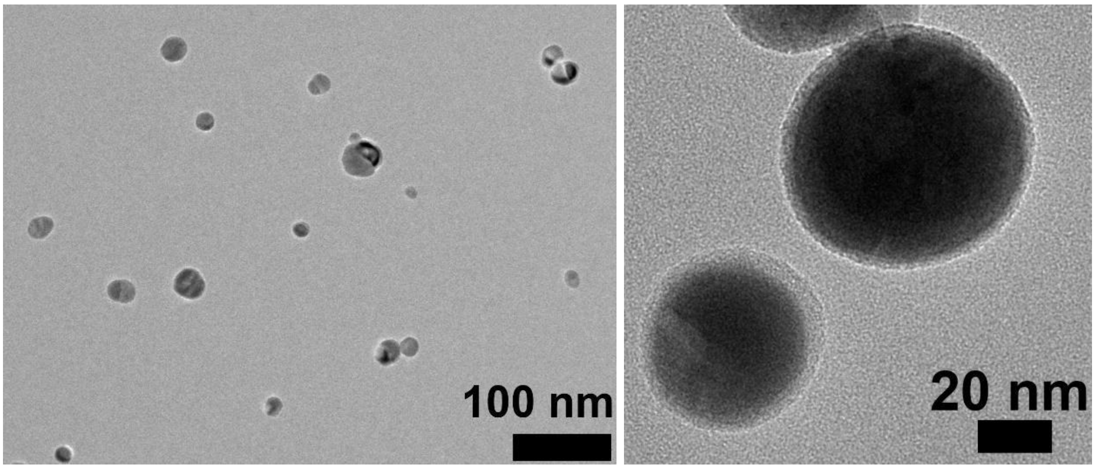

Figure S3. TEM images of the individual Ge/Sn nanoparticles with crystal core covering by amorphous shell, prepared in spark discharge with additional heat treatment in a tube furnace at 750 °C.
